# Des-acyl ghrelin reduces alcohol intake and alcohol-induced reward in rodents

**DOI:** 10.1038/s41398-024-02996-8

**Published:** 2024-07-04

**Authors:** Sarah Witley, Christian E. Edvardsson, Cajsa Aranäs, Maximilian Tufvesson-Alm, Darta Stalberga, Henrik Green, Jesper Vestlund, Elisabet Jerlhag

**Affiliations:** 1 Department of Pharmacology, Institute of Neuroscience and Physiology, The Sahlgrenska Academy at the University of Gothenburg, Gothenburg, Sweden; 2 Division of Clinical Chemistry and Pharmacology, Department of Biomedical and Clinical Sciences, Faculty of Medicine, Linköping University, Linköping, Sweden; 3 Department of Forensic Genetics and Forensic Toxicology, National Board of Forensic Medicine, Linköping, Sweden

**Keywords:** Addiction, Neuroscience

## Abstract

The mechanisms contributing to alcohol use disorder (AUD) are complex and the orexigenic peptide ghrelin, which enhances alcohol reward, is implied as a crucial modulator. The major proportion of circulating ghrelin is however the non-octanoylated form of ghrelin, des-acyl ghrelin (DAG), whose role in reward processes is unknown. As recent studies show that DAG decreases food intake, we hypothesize that DAG attenuates alcohol-related responses in animal models. Acute and repeated DAG treatment dose-dependently decreased alcohol drinking in male and female rats. In these alcohol-consuming male rats, repeated DAG treatment causes higher levels of dopamine metabolites in the ventral tegmental area, an area central to reward processing. The role of DAG in reward processing is further supported as DAG prevents alcohol-induced locomotor stimulation, reward in the conditioned place preference paradigm, and dopamine release in the nucleus accumbens in male rodents. On the contrary, DAG does not alter the memory of alcohol reward or affect neurotransmission in the hippocampus, an area central to memory. Further, circulating DAG levels are positively correlated with alcohol drinking in female but not male rats. Studies were conducted in attempts to identify tentative targets of DAG, which currently are unknown. Data from these recombinant cell system revealed that DAG does not bind to either of the monoamine transporters, 5HT2A, CB1, or µ-opioid receptors. Collectively, our data show that DAG attenuates alcohol-related responses in rodents, an effect opposite to that of ghrelin, and contributes towards a deeper insight into behaviors regulated by the ghrelinergic signaling pathway.

## Introduction

Alcohol use disorder (AUD) is a major public health problem with high morbidity and mortality [1]. Its development is complex and the dopaminergic neurons from the ventral tegmental area (VTA) to the nucleus accumbens (NAc) have gained extra interest as they mediate alcohol-induced reward (for review see [2, 3]). Various biological systems participate in the ability of alcohol to interact with these dopamine neurons, and gut-brain peptides have been implied as important modulators (for review see [4]). One of these peptides is ghrelin (i.e., acyl-ghrelin, the acylated form of ghrelin [5]), an orexigenic peptide with profound effects on alcohol-related behavior (for review see [4]). Both preclinical and clinical studies report that ghrelin enhances, whereas suppression of the ghrelin receptor (GHSR) reduces alcohol-related responses [6–18]. Intriguingly, preliminary data report that a GHSR inverse agonist reduces alcohol craving in patients with AUD [19, 20]. Moreover, polymorphisms of the *GHSR* gene are associated with different aspects of AUD, a finding also true for the *pre-pro-ghrelin* gene [21–25]. The notion that the *pre-pro-ghrelin* gene encodes for both ghrelin and des-acyl-ghrelin (DAG), the main proportion of circulating ghrelin, raises the possibility that DAG may influence alcohol responses.

DAG can be found in the plasma after ghrelin is secreted from the gastrointestinal tract and subsequently deacetylated in the serum [26]. DAG was previously considered to be inactive [27–31], but has been found to decrease food intake [32]. Moreover, DAG blocks the effects of ghrelin on feeding, gastric emptying, body temperature, and glucose and lipid metabolism [30–34]. Based on these findings and given that ghrelin has a prominent role in mediating alcohol-related responses, we hypothesize that DAG decreases such effects in rodents.

To evaluate this hypothesis, we tested if an acute DAG injection dose-dependently reduces alcohol intake in male and female rats. Additional experiments of rats of both sexes explored the possibility that repeated DAG treatment decreases alcohol consumption. To elucidate a process underlying the decrease in alcohol intake by DAG, the monoamine levels were measured in various reward-related areas from these rats. As DAG alters dopaminergic transmission in the VTA, an area modulating reward, the effect of DAG on different reward paradigms, including alcohol-induced locomotor stimulation, conditioned place preference (CPP), and dopamine release in NAc shell, was tested. To explore the interaction with dopamine further, we explored if DAG inhibits the dopamine transporter (DAT) expressed in a recombinant cell system. Additional studies explored the possibility that DAG acts via other systems specifically the transporter of serotonin (SERT) or noradrenaline (NET) or activates the 5HT2A, CB1, or µ-opioid receptors. Finally, the serum levels of DAG in low- and high-alcohol preferring rats of both sexes were measured. Collectively, these data will fill an unidentified knowledge gap regarding DAG and alcohol-related responses in rodents.

## Materials and methods

### Animals

Male and female Rcc Han Wistar rats (8–12 weeks old at arrival; Envigo, Horst, Netherlands) were used for the alcohol drinking and locomotor activity experiments as they drink alcohol to intoxicating levels [35]. For in vivo microdialysis studies male Wistar rats (Charles River, Calco, Italy) were used as they display robust dopamine levels in NAc shell after alcohol injection [36]. For the behavioral tests, male mice of the NMRI (8–12 weeks old at arrival; Charles River, Sulzfeld, Germany) strain were used as it shows robust responses to alcohol in these models [37, 38]. All rodents were allowed to habituate to the animal facility at least one week before they entered an experiment. During housing (20 °C with 50% humidity) the rodents had free access to water and standard chow (Teklad Rodent Diet; Envigo, Madison, WI, USA). These experiments were approved by the Ethics Committee for Animal Experiments (ethical numbers: 1457/18, 3276/18, 1556/18, 3348/20; Gothenburg, Sweden) and followed the ARRIVE guidelines. The sample sizes of each experiment were based on previous experience in which statistical significance has been detected. The person who conducted the experiments, and analyzed the collected data was aware of the treatment allocation. Only preset exclusion criteria were used when analyzing the data obtained in attempts to exclude confounding factors in analyzing data. In the alcohol drinking experiments leaking bottles and low alcohol consumption (<3.0 g/kg for males; <3.5 g/kg for females) were preset exclusion criteria. The preset exclusion criterion in the ex vivo HPLC-EC, ELISA, and recombinant cell line experiments was contamination of the sample. Misplaced probes, technical difficulties, and damages to the surgery area were preset exclusion criteria for the in vivo microdialysis experiment. Intra-cage male aggression was the preset exclusion criterion for the locomotor activity and CPP experiments. To minimize tentative confounding factors, the order of treatments was stratified, with treatments being mixed for each animal cage and for each apparatus used as well as balancing the alcohol intake between future treatment groups. Although each experiment only was performed once, they were separated over different laboratory days with equal data obtained between sessions.

### Drugs

Alcohol (95%; Solveco AB, Stockholm, Sweden) was either diluted to a 20% solution using tap water (drinking experiment) or to a 15% (w/v) solution with 0.9% NaCl. Alcohol was in the behavioral and neurochemical tests injected (intraperitoneally, IP) 5 min before the test. A dose of 1.75 g/kg was used in male mice as it causes robust locomotor stimulation and induces a CPP [37–39], and 2 g/kg was used for rats as it elevates the dopamine levels in NAc shell [40, 41]. DAG (Tocrise Bioscience, Bristol, UK) was dissolved in 0.9% NaCl in doses (0.25–2 mg/kg) known to reduce food intake [32] and DAG was injected IP).

### Experiments

#### Effects of an acute or repeated DAG treatment on alcohol intake in female and male rats

Individually housed rats had intermittent access to an alcohol (20%) bottle and a water bottle, three 24-h sessions per week, and only water bottles the remaining days [35]. A reversed light/dark cycle was used, and the bottles were changed when the lights went out. First, the rats consumed alcohol for 10 weeks (baseline drinking), where the bottles and food were weighed daily and the body weight weekly. During treatments, the bottles and food were weighed at 4- and/or 24-h and the body weight was measured daily. In the first drinking experiment, the potential of DAG to reduce alcohol consumption was tested and therefore, vehicle or DAG (1 mg/kg) was acutely injected into male (*n* = 12) or female (*n* = 12) rats. The second drinking experiment was conducted to establish the effects of lower DAG doses on alcohol intake. Therefore, separate male (*n* = 18) or female (*n* = 18) rats, were acutely injected with lower DAG doses (0.5 or 0.25 mg/kg) or vehicle. The third alcohol drinking experiment evaluated the ability of repeated DAG injections to lower alcohol drinking. Therefore, additional male (*n* = 24) and female (*n* = 24) rats were injected with either vehicle or DAG (1 mg/kg) at three alcohol-drinking sessions. At the end of the third drinking session, the rats were euthanized and decapitated, and the brains were placed in a brain-slicing matrix. Brain areas central for reward (VTA, NAc shell, NAc core, LDTg, dorsal striatum, hippocampus, paraventricular thalamus, prefrontal cortex, central amygdala) were punched and stored at −80 °C. Each sample was homogenized, and then the monoamine content in the supernatant was analyzed in an HPLC-EC system as described before [42], allowing tentative determination of brain regions, directly or indirectly, affected by DAG.

Another set of rats voluntarily consumed alcohol for 12 weeks before division into low- and high-consumers (cut-off >3 g/kg for males and >4 g/kg for females). As the *pre-pro-ghrelin* gene encodes for DAG, ghrelin, and obestatin, the effects of long-term alcohol consumption on the serum levels of DAG, total ghrelin (ghrelin+DAG), and obestatin were evaluated in rats of both sexes. The alcohol-drinking male (*n* = 22) and female (*n* = 22) rats were sacrificed, and trunk blood was collected into serum tubes (10 mL *95 × 16.8* *mm Z-gel*, Sarstedt, Helsingborg, Sweden) that were shaken and incubated for at least 30 min in line with the manufacturer’s instructions. After centrifugation (4600 rpm, 10 min, RT), the concentration of DAG (NBP2-82459, Novus Biologicals, Bio-Techne, Abingdon, United Kingdom), total ghrelin (EZRGT, Sigma-Aldrich, Merck, Darmstadt, Germany) or obestatin (RAB0208, Sigma-Aldrich) was measured in serum by ELISA kits. A microplate photometer (Multiskan GO, Thermo Fisher Scientific, Darmstadt, Germany) at a defined wavelength (DAG, 450 nm; total ghrelin, 450 nm obestatin, 450 nm) was used to detect the fluorescence intensity of each duplicate serum sample placed on the multi-well plates.

#### Effects of DAG treatment on reward-associated behaviors and neurochemistry in male rodents

Locomotor activity tests were conducted in ventilated and dim-lit (4 lux) open field arenas (420 × 420 × 200 mm; Med Associates Inc; Georgia, Vermont, USA). Infra-red beams detected distance traveled, number of jumps, vertical counts, average velocity, stereotypic counts, number of inner zone entries, and time spent in the inner/outer zones of each animal.

Initial locomotor activity tests of rats and mice were conducted to define a dose of DAG that does not alter locomotor activity per se. In the first test, after 60 min of habituation, male (*n* = 24) or female (*n* = 24) rats were injected with vehicle or DAG (0.5, 1.0, or 2.0 mg/kg). 15 min later the activity was monitored for another 60 min. In the second locomotor activity experiment, male mice (*n* = 32) were allowed to habituate for 60 min. After that, they were injected with vehicle or DAG (0.25, 0.5, or 1.0 mg/kg). After 15 min the activity was monitored for another 60 min.

Additional locomotor activity experiments were conducted, where the ability of DAG to dose-dependently block the alcohol-induced locomotor stimulation was explored. Therefore, male mice were (*n* = 32) injected with DAG (1.0 mg/kg) or vehicle 15 min before alcohol or vehicle. The activity registration started 5 min later and was recorded for an additional 60 min. Identical locomotor activity experiments were conducted in new male mice (*n* = 32), where a lower dose of DAG (0.5 mg/kg) was combined with alcohol.

In the CPP paradigm, the effect of DAG (1.0 mg/kg) on alcohol reward (rCPP) or memory of alcohol reward (mCPP) was explored in male mice. As described before [37, 38], a two-chambered CPP apparatus (3 lux) with distinct tactile/visual cues was used. The paradigm consists of pre-conditioning (day 1), conditioning (days 2–5), and post-conditioning (day 6), where each session was 20 min. In the rCPP paradigm, male mice (*n* = 16) were untreated at pre-conditioning and were allowed to explore both CPP compartments. The mice were then conditioned to vehicle-alcohol or DAG-alcohol on the least preferred side (biased design). The mice were untreated at post-conditioning and were allowed to explore both CPP compartments, creating the following treatment groups: DAG-alcohol and vehicle-alcohol. In the mCPP paradigm, male mice (*n* = 16) received a pre-test injection of vehicle. Subsequently, they were allowed to freely explore both compartments of the CPP setup. The mice were then conditioned to alcohol on the least preferred side (biased design) at each conditioning day. At post-conditioning the mice were injected with vehicle or DAG and were allowed to explore both CPP compartments, creating the following treatment groups: alcohol-DAG and alcohol-vehicle. Two control experiments were conducted in additional male mice to exclude the possibility that DAG affects rCPP (*n* = 16) or mCPP (*n* = 16) per se. They were conducted similarly, but only the vehicle was injected during conditioning. CPP was defined as the time at least preferred compartment at post-conditioning minus pre-conditioning divided by the total CPP time.

In vivo microdialysis experiments were used to test the hypothesis that DAG blocks alcohol-induced dopamine release in the NAc shell. A probe was through surgery placed in NAc shell (+1.85 AP, ±1.0 mm LM, and 7.4 mm VP [43]) of male rats (*n* = 32) as described before [37–39]. Three days later, the rats were connected to the microdialysis apparatus and allowed to habituate for 60 min before three baseline samples were collected. Thereafter, the rats received an acute injection with DAG (1.0 mg/kg) or vehicle followed by an injection with alcohol or vehicle. After treatments, 10 additional samples were collected. After the termination, the probe location was determined, and only rats with correct placements were included in the statistical analysis.

DAG may indirectly or directly influence the VTA to NAc projection as the present in vivo microdialysis studies showed that DAG prevented the alcohol-induced dopamine release in NAc shell and that DAG altered the ex vivo levels of neurotransmitters in the VTA of alcohol-drinking male rats. We therefore explore if DAG influenced the activity of neurons (cFOS) in NAc and VTA. Naïve male rats were treated acutely with vehicle (*n* = 3) or DAG (*n* = 3) and were 90 min later perfused [42, 44]. Brains were collected, and stored in 4% paraformaldehyde overnight, and in 25% sucrose until sectioning. Brains were sectioned coronally in 40-μm sections using a microtome. Brain slices of NAc (AP +1.8 mm) and VTA (AP −5.2 mm) were stained for cFOS-DAB, as described elsewhere [42, 44], visualized by a Nikon light microscope (Nikon Eclipse 90i, Tokyo, Japan). Pictures were captured with a Nikon DS-Fi1 camera (Nikon, Tokyo, Japan). Cell bodies positive for cFOS immunoreactivity (dark brown) were quantified using ImageJ-FIJI (National Institutes of Health, Bethesda, MD), and counted manually by a blinded experimenter.

To explore the interaction between DAG and dopamine further, we investigated if DAG (top dilution: 8.3 µM) inhibits the human transporter of dopamine (DAT) in a recombinant cell line (CHO-K1 cells from PerkinElmer; Hagersten, Sweden). The tentative inhibition of DAG to SERT and NET was then tested. The transporter activity assessment was performed according to a published method [45], with slight adjustments. In short, 50,000 nM cocaine was used as a positive control, and drug-serial dilutions (1:2) in 15 steps were prepared using a QIAgility work robot (Qiagen, Kistan, Sweden). Each well contained 12.5 ml of the compound, 12.5 ml of dye mix (neurotransmitter transporter uptake assay kit, Molecular Devices, Wokingham, UK), and 50 ml cells (0.3 million cells/ml). After 3 h incubations for all cell lines, the fluorescence was measured on a TECAN Spark 10 M (Tecan, Stockholm, Sweden) with the following settings: manual gain—80; Z-position—20,000; excitation wavelength—430 nm; emission wavelength—535 nm; multiple reads per well (3 × 3); and 10 flashes.

DAG activity was also screened towards three human receptors serotonin (5HT2A), cannabinoid (CB1), and µ-opioid using an in vitro Aquoscreen® assay previously published by [46]. In short, 70 µM of LSD, 60 µM of JWH-018, and 60 µM of fentanyl were used as control compounds for screening on 5HT2A, CB1, and µ-opioid accordingly and tested with 8.3 µM DAG. Digitonin (100 µM) and ATP (10 µM) were used as positive controls. All test compounds were prepared in an 8–10 step dilution series of 1:8 and exposed to a recombinant cell line expressing the target receptor in triplicates. The luminescence was read using TECAN Spark 10 M (Tecan, Switzerland) in a total of 200 reads/well, and cell addition on the plate (~15,000 cells/well) after the first 10 baseline reads.

### Statistical analysis

The obtained data are normally distributed and with equal variance. The data from drinking experiments were analyzed by unpaired t-test or repeated two-way ANOVA. A two-tailed unpaired t-test analyzed the ELISA, ex vivo HPLC-EC, and cFOS data. In addition, the Pearson test correlated the serum levels and the mean values of alcohol intake in rats. A one-way ANOVA with Bonferroni post hoc tests and unpaired *t*-test analyzed the locomotor activity and CPP data, respectively. The in vivo microdialysis experiments were evaluated by a repeated two-way ANOVA followed by the Bonferroni post hoc test. All data were adjusted for multiple testing. The EC50 (half maximal effective concentration) and non-parametric curve fittings (three parameters) were calculated for the recombinant cell line data. Data were analyzed with GraphPad Prism version 10.0.2 (GraphPad Software, La Jolla, CA, USA).

## Results

### Acute and repeated DAG treatment reduced alcohol intake in male and female rats

**Fig. 1:**
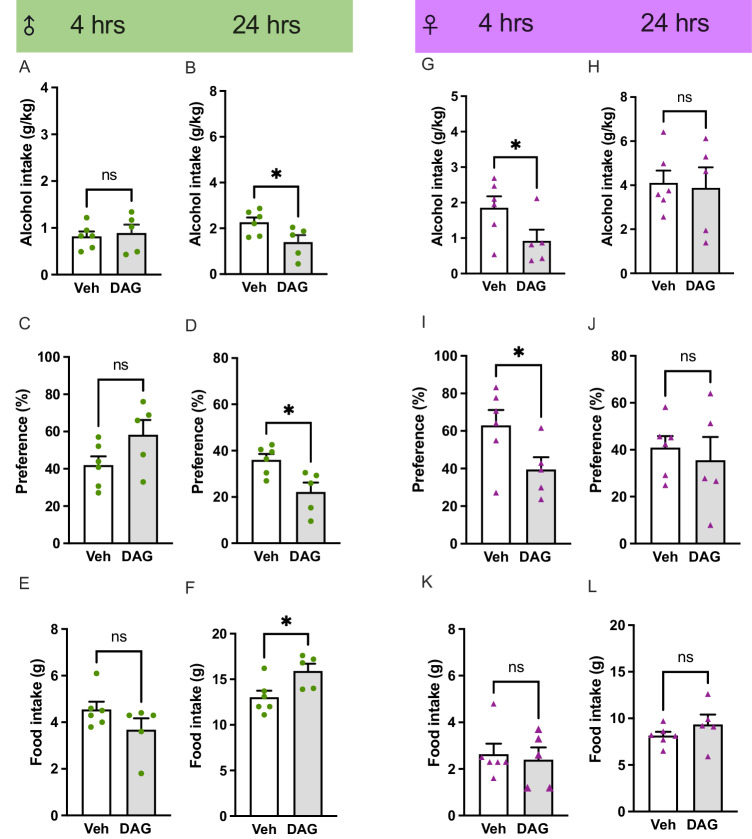
Effects of acute DAG treatment in alcohol-drinking male and female rats. In male rats later exposed to an acute injection of DAG (1.0 mg/kg IP; 4.3 ± 0.5 g/kg; *n* = 5) or vehicle (3.9 ± 1.3 g/kg; *n* = 6) the average alcohol intake during baseline was similar (t_9_ = 0.60; *P* = 0.2803). In these male rats, an acute DAG injection did not alter the **A** 4-h alcohol intake (t_9_ = 0.36; *P* = 0.7264), while it **B** reduced the alcohol intake at the 24-h time point (t_9_ = 2.35; *P* = 0.0432). DAG did not alter the **C** preference for alcohol at the 4-h time point (t_9_ = 1.84; *P* = 0.0983), **D** but decreased it at 24 h (t_9_ = 3.00; *P* = 0.0149). While DAG did not affect the **E** 4-h food intake (t_9_ = 1.51; *P* = 0.1657), **F** it increased the 24-h feeding (t_9_ = 2.64; *P* = 0.0271). In female rats later exposed to an acute DAG (7.3 ± 0.8 g/kg; *n* = 5) or vehicle (6.3 ± 0.8 g/kg; *n* = 6) injection the baseline alcohol intake did not differ (t_9_ = 0.69; *P* = 0.5049). While an acute DAG injection **G** decreased the 4-h alcohol intake (t_9_ = 2.05; *P* = 0.0351) in these female rats, **H** it did not alter the 24-h drinking (t_9_ = 0.22; *P* = 0.8331). When it comes to the preference for alcohol, DAG reduced it at the **I** 4-h (t_9_ = 2.16; *P* = 0.0294), **J** but not the 24-h time point (t_9_ = 0.52; *P* = 0.6183). At neither time point, DAG affected the **K**, **L** food intake (t_9_ = 0.34; *P* = 0.7414 and t_9_ = 1.12; *P* = 0.2909, respectively). Data are presented as mean ± SEM, significant data are illustrated by *P < 0.05, n.s. *P* > 0.05. Vehicle (Veh), Des-acyl-ghrelin (DAG).

In the male rats, an acute DAG injection did not alter the 4-h alcohol intake or alcohol preference, whereas it decreased both parameters (*P* = 0.0432 and *P* = 0.0149, respectively) at the 24-h time point (Fig. 1A–D). While DAG did not affect the 4-h food intake in males, it increased the 24-h feeding (*P* = 0.0271; Fig. 1E, F). In female rats, an acute DAG injection decreased the 4-h alcohol intake (*P* = 0.0351) and alcohol preference (*P* = 0.0294), but it did not alter the 24-h values (Fig. 1G–J). The food intake was not affected by DAG at any time point in females (Fig. 1K, L). In both male and female rats, the water and total fluid intake as well as body weight was similar between DAG- and vehicle-treated rats (Supplementary Fig. 1A–J). One male (<3.0 g/kg) and one female (<3.5 g/kg) rat were excluded due to low baseline alcohol intake.

Acute injection of lower DAG doses (0.5 or 0.25 mg/kg) did not alter the intake of alcohol, water, total fluid, food intake alcohol preference or body weight in male or female rats (Supplementary Fig. 2A–W; *n* = 6 for all treatment groups, but DAG (0.5 mg/kg) treated males (*n* = 5) as one rat was excluded due to low baseline alcohol intake, <3.0 g/kg).

**Fig. 2:**
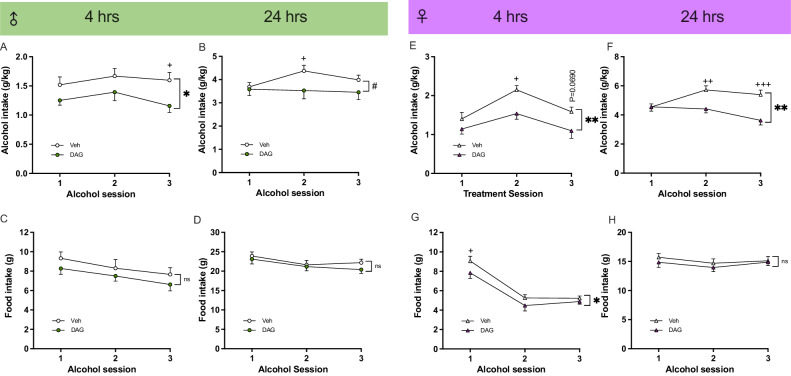
Effects of repeated DAG treatment in alcohol-drinking male and female rats. Male rats treated repeatedly with vehicle (4.0 ± 0.2 g/kg; *n* = 8) or DAG (3.7 ± 0.2 g/kg; *n* = 8), had a similar alcohol intake during baseline (t_14_ = 1.22; *P* = 0.2423). Repeated DAG injections had an overall decrease in alcohol intake at the **A** 4-h time point (treatment F_1,14_ = 5.12, *P* = 0.0401 *; time F_2,28_ = 1.87, *P* = 0.1754; interaction F_2,28_ = 0.59, *P* = 0.5622), and the alcohol intake was specifically lower after DAG at alcohol drinking session 3 (*P* = 0.0256). **B** An overall reduction in alcohol intake was observed after DAG injections (treatment F_1,14_ = 1.96, *P* = 0.1836; time F_2,28_ = 3.44, *P* = 0.0460; interaction F_2,28_ = 4.34, *P* = 0.0228 #), specifically at alcohol drinking session 2 (*P* = 0.0324). **C**, **D** Repeated DAG injections did not alter the food intake at any time point (4-h: treatment F_1,14_ = 1.41, *P* = 0.2546; time F_2,28_ = 6.86, *P* = 0.0045; interaction F_2,28_ = 0.05, *P* = 0.9518; 24-h: treatment F_1,14_ = 0.55, *P* = 0.4727; time F_2,28_ = 13.79, *P* < 0.0001; interaction F_2,28_ = 1.03, *P* = 0.3707). The alcohol consumption was similar in female rats later treated with vehicle or DAG (t_14_ = 0.62; *P* = 0.5477; vehicle, 5.2 ± 0.4 g/kg; *n* = 9; or DAG, 4.9 ± 0.3 g/kg; *n* = 7). **E** In these female rats, repeated DAG injections overall reduced the alcohol consumption at the 4-h time point (treatment F_1,14_ = 9.17, *P* = 0.0090 **; time F_2,28_ = 12.63, *P* = 0.0001; interaction F_2,28_ = 1.02, *P* = 0.3754), a decrease specifically evident at alcohol drinking session 2 (*P* = 0.0158). **F** An overall decline in 24-h alcohol intake was observed (treatment F_1,14_ = 14.72, *P* = 0.0018 **; time F_2,28_ = 2.91, *P* = 0.0711; interaction F_2,28_ = 6.43, *P* = 0.0051 ##), specifically at alcohol-drinking sessions 2–3 (*P* = 0.0063 and *P* = 0.0002, respectively). **G** A lowered food intake by repeated DAG injections was observed at the 4-h (treatment F_1,14_ = 4.80, *P* = 0.0460 *; time F_2,28_ = 4.79, *P* < 0.0001; interaction F_2,28_ = 0.57, *P* = 0.5742), **H** but not at the 24-h time point (treatment F_1,14_ = 0.53, *P* = 0.4798; time F_2,28_ = 1.68, *P* = 0.2049; interaction F_2,28_ = 0.19, *P* = 0.8269). The lowered food intake by DAG was found at alcohol drinking session 2 (*P* = 0.0475). Data are presented as mean ± SEM. *P < 0.05, **P < 0.01 shows an overall treatment effect. #*P* < 0.05, shows an overall interaction effect. Significant differences between the vehicle (Veh) and des-acyl-ghrelin (DAG) for a specific time point are illustrated by +*P* < 0.05, ++*P* < 0.01, +++*P* < 0.001.

In male rats, repeated DAG injections overall decreased the alcohol intake at 4- (*P* = 0.0401) and 24-h (*P* = 0.0228) time points, and this reduction was evident at alcohol drinking session 3 (*P* = 0.0256) and 2 (*P* = 0.0324), respectively (Fig. 2A, B). Repeated DAG injections did not affect the food intake in males (Fig. 2C, D). In females, repeated DAG injections overall reduced the alcohol consumption at the 4-h time point (*P* = 0.0090), a decrease specifically evident at alcohol drinking session 2 (*P* = 0.0158) (Fig. 2E). Furthermore in females, DAG injections lowered the 24-h alcohol intake (*P* = 0.0018), specifically at alcohol-drinking sessions 2–3 (*P* = 0.0063 and *P* = 0.0002, respectively) (Fig. 2F). Repeated DAG injections overall lowered the food intake at the 4-h time point (*P* = 0.0460) and specifically at alcohol drinking session 2 (*P* = 0.0475) (Fig. 2G). On the contrary, it did not influence 24-h feeding (Fig. 2H). In male rats, the preference for alcohol, water intake, and body weight were unchanged after repeated DAG injections, whereas DAG treatment hand an overall decline in the total fluid intake at the 24-h time point (*P* = 0.0304) (Supplementary Fig. 3A–G). In females, the alcohol preference, the water intake, and the total fluid intake remained unaltered by repeated injections of DAG (Supplementary Fig. 3H–M). The female’s body weight was not affected by repeated DAG treatment (Supplementary Fig. 3N). Due to low baseline alcohol intake, 8 males (<3.0 g/kg) and 8 females (3.5 g/kg) were excluded.

### In alcohol-drinking males, but not females, repeated DAG treatment alters dopamine signaling in the VTA

**Fig. 3:**
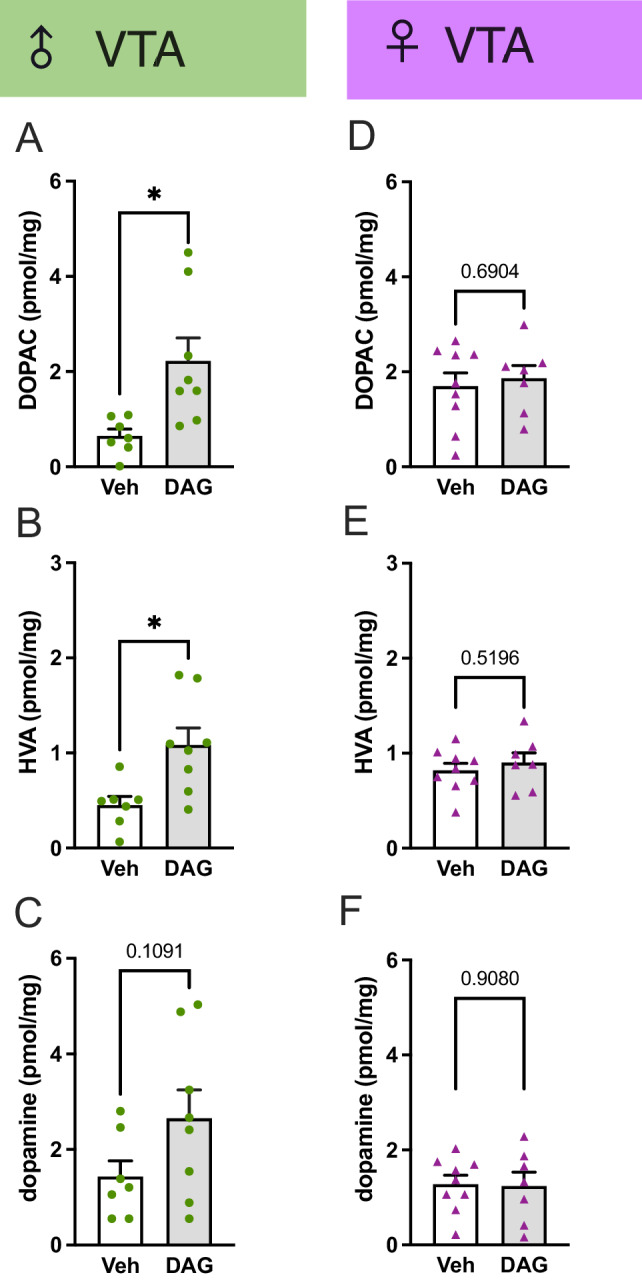
Effects of repeated DAG on neurotransmission in the VTA in alcohol-drinking male and female rats. In the alcohol-drinking male rats repeated DAG injections increased the ex vivo levels of **A** DOPAC (t_13_ = 2.94, *P* = 0.0116) and **B** HVA (t_13_ = 3.00, *P* = 0.0102) and **C** tended to elevate the dopamine levels (t_13_ = 1.72, *P* = 0.1091). On the contrary, repeated DAG treatment did not change the levels of **D** DOPAC (t_14_ = 0.41, *P* = 0.6904), **E** HVA (t_14_ = 0.66, *P* = 0.5196), or **F** dopamine (t_14_ = 0.12, *P* = 0.9080) in the VTA of alcohol-drinking female rats. Data are presented as mean ± SEM. A significant difference between vehicle (Veh) and des-acyl-ghrelin (DAG) is shown by *P < 0.05.

In alcohol-drinking male rats, repeated DAG reduced dopaminergic neurotransmission in the VTA. Compared to vehicle (*n* = 7, one sample excluded due to contamination), repeated DAG (*n* = 8) injections increased the ex vivo levels of 3,4-Dihydroxyphenylacetic acid (DOPAC, *P* = 0.0116, Fig. 3A) and homovanillic acid (HVA, *P* = 0.0102, Fig. 3B) and tended to enhance the dopamine levels (*P* = 0.1091, Fig. 3C). In alcohol-drinking female rats, repeated DAG (*n* = 7) treatment did not change the levels of DOPAC, HVA, or dopamine in the VTA compared to vehicle (*n* = 9) (Fig. 3D–F). In these alcohol-drinking male or female rats, repeated DAG treatment neither affected the dopamine turnover, 5-hydroxy indole acetic acid (5-HIAA), serotonin, nor serotonin turnover (Supplementary Fig. 4A–D, F–I). Moreover, DAG treatment tended to elevate the ex vivo noradrenaline levels in this area of male rats (Supplementary Fig. 4E, *P* = 0.0819), but not in female rats (Supplementary Fig. 4J). Neither in males nor females, DAG altered the monoaminergic neurotransmission in the central amygdala, hippocampus, laterodorsal tegmental area, NAc core and NAc shell, prefrontal cortex, paraventricular thalamus, striatum (data not shown as treatment did not influence these parameters).

In male rats, DAG did not alter the number of cFOS-positive cells in the NAc shell (237 ± 13 for vehicle, and 219 ± 33 for DAG, t_4_ = 0.60, *P* = 0.6372, *n* = 3 per treatment group, Supplementary Fig. 5A, B). Neither did DAG treatment influence cFOS-positive cells in the VTA (data not shown).

### DAG reduced reward-related responses in male rodents

**Fig. 4:**
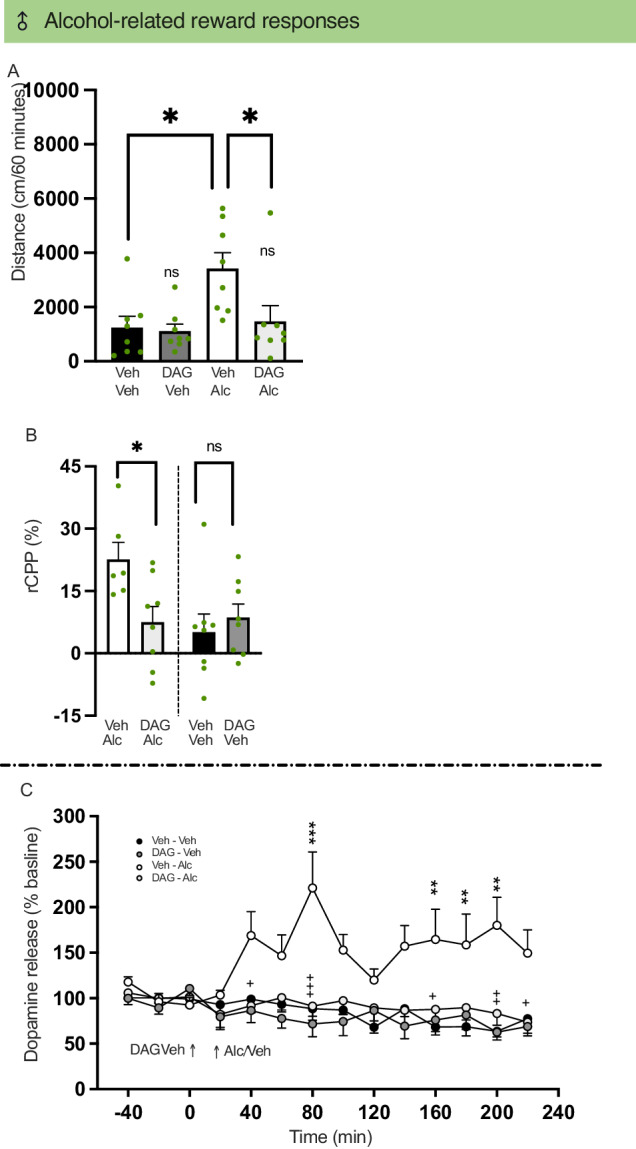
Effects of acute DAG treatment on reward-related parameters in males. **A** Treatment had an overall effect on locomotor activity in male mice (F_3,28_ = 5.08, *P* = 0.0062). Compared with vehicle, alcohol caused a locomotor simulation (*P* = 0.0136). Acute treatment with des-acyl-ghrelin (DAG; 1 mg/kg), prevented the alcohol-induced locomotor stimulation (*P* = 0.0305). The activity level was similar between vehicle and DAG-alcohol-treated mice (*P* > 0.9999). DAG did not alter the activity of the mice (*P* > 0.9999). **B** DAG (1 mg/kg) attenuated the reward of alcohol in the conditioned place preference test (rCPP, t_12_ = 2.69, *P* = 0.0197) in male mice. Moreover, DAG did not influence rCPP (t_14_ = 0.64, *P* = 0.5304) per se. **C** In the in vivo microdialysis experiment, treatment had an overall effect on dopamine release in the nucleus accumbens (NAc) shell of male mice (treatment F_3,28_ = 13.93, *P* < 0.0001; time F_13,364_ = 1.29, *P* = 0.2140; interaction F_39,364_ = 2.60, *P* < 0.0001). Specifically, alcohol elevated dopamine in NAc shell (80 min *P* < 0.001, 160 min *P* = 0.0011, 180 min *P* = 0.0037, 200 min *P* < 0.0001) and DAG (1 mg/kg) suppressed this enhancement (40 min *P* = 0.0396, 80 min *P* < 0.0001, 160 min *P* = 0.0400, 200 min *P* = 0.0010, 220 min *P* = 0.0458). The dopamine levels were similar between vehicle and DAG-alcohol-treated mice. Compared to vehicle (Veh), DAG did not affect dopamine in the NAc shell per se. Data are presented as mean ± SEM. Significant difference is shown by *P < 0.05, **P < 0.01, ***P < 0.001. Significant differences between vehicle-alcohol and DAG-alcohol for a specific time point are illustrated by +*P* < 0.05, ++*P* < 0.01, +++*P* < 0.001.

Treatment had an overall effect on locomotor activity in male mice (*P* = 0.0062). Specifically, the alcohol-induced locomotor stimulation (*P* = 0.0136) was blocked by DAG (1 mg/kg, *P* = 0.0305) (Fig. 4A, *n* = 8 per treatment group). Similarly, there was an overall effect on locomotor activity by a lower DAG dose (0.5 mg/kg) and alcohol (*P* = 0.0334). The lower DAG dose reduced but did not block (*P* = 0.5319), this hyperlocomotion after alcohol (*P* = 0.0225) (Supplementary Fig. 6A, *n* = 8 per treatment group, but vehicle-vehicle group where one was removed due to intra-cage fighting before the test). While DAG (1 mg/kg) attenuated the reward of alcohol in the CPP test (rCPP, t_12_ = 2.69, *P* = 0.0197, Fig. 4B), it did not reduce the memory of alcohol reward (mCPP, t_14_ = 1.36, *P* = 0.1955) (Supplementary Fig. 6B, *n* = 8 per treatment group, but the vehicle-alcohol group (*n* = 7) where one mouse was removed to intra-cage fighting before test). In additional experiments, treatment had an overall effect on dopamine release in the NAc shell of male mice (*P* < 0.0001). Specifically, alcohol elevated dopamine in NAc shell (80 min *P* < 0.001, 160 min *P* = 0.0011, 180 min *P* = 0.0037, 200 min *P* < 0.0001) and DAG (1 mg/kg) suppressed this enhancement (40 min *P* = 0.0396, 80 min *P* < 0.0001, 160 min *P* = 0.0400, 200 min *P* = 0.0010, 220 min *P* = 0.0458) (Fig. 4C, *n* = 8 per treatment group). Six rats were excluded due to misplaced probes. DAG did neither influence locomotor activity, rCPP, mCPP nor accumbal dopamine per se (Fig. 4 and Supplementary Fig. 6).

### Female rats displayed a correlation between DAG in serum and alcohol intake

**Fig. 5:**
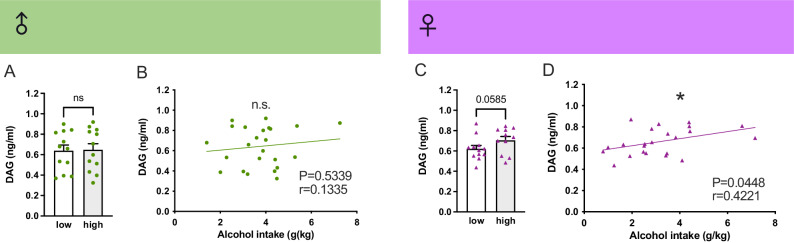
Female rats display a correlation between DAG in serum and alcohol intake. **A** There was no difference in serum des-acyl-ghrelin (DAG) between male rats consuming low (*n* = 12) or high (*n* = 12) amounts of alcohol (t_22_ = 0.11; *P* = 0.9137). **B** Besides, the DAG levels did not correlate with alcohol consumption in male rats (r_22_ = 0.1335, *P* = 0.53399). **C** Contrarily, high alcohol-consuming female rats (*n* = 11) tended to display higher amounts of serum DAG (t_21_ = 1.64; *P* = 0.0585) compared to low alcohol-consuming female rats (*n* = 12). **D** This difference was further evident as DAG concentrations positively correlated with alcohol consumption in females (r_21_ = 0.4221; *P* = 0.0448). One sample from the high alcohol-consuming female rats was excluded due to contamination.

There was no difference in serum DAG between male rats consuming low (*n* = 12) or high (*n* = 12) amounts of alcohol (Fig. 5A), and there was no correlation between the two (Fig. 5B). High alcohol-consuming female rats (*n* = 11) tended to display higher amounts of serum DAG (*P* = 0.0585; Fig. 5C) than low alcohol-consuming rats (*n* = 12), and the DAG concentrations positively correlated with alcohol consumption (*P* = 0.0448; Fig. 5D). No difference in total ghrelin was observed between high and low-alcohol-consuming males (Supplementary Fig. 7A), and the serum levels did not correlate to alcohol intake (Supplementary Fig. 7B). A similar finding was displayed in female rats (Supplementary Fig. 7C, D). No difference in obestatin was observed between low and high-alcohol-consuming male rats (Supplementary Fig. 7E), and no correlation between obestatin and alcohol intake was observed (Supplementary Fig. 7F). Neither was there a correlation between obestatin and alcohol in female rats (Supplementary Fig. 7G, H). Due to sample contamination, one sample from the high alcohol-consuming female rats w was excluded.

### Effects on DAG activity towards monoamine transporters and receptors in recombinant cell lines

DAG was tested for inhibition towards dopamine (DAT), norepinephrine (NET), serotonin (SERT) transporters, and activity towards 5HT2A, CB1, and µ-opioid receptors. In the transporter inhibition assay, 8.3 µM DAG showed no inhibition of DAT, NET, or SERT (Supplementary Fig. 8A). While the reference antagonist, cocaine, was tested at a top concentration of 50 µM, even further dilution series showed full NET and SERT inhibition at 12.5 µM and 6.25 µM, while DAT inhibition at such concentration reached 70–80% of full transporter inhibition (Supplementary Fig. 8C, D). In addition, 50 µM DAG showed no activation of 5HT2A, CB1, or µ-opioid receptor (Supplementary Fig. 8B). Reference compounds 70 µM LSD, 60 µM JWH-018, and 60 µM of fentanyl showed full target receptor inhibition also at 8.75 µM, 7.5 µM, and 7.5 µM, respectively, whereas DAG did not (Supplementary Fig. 8E–G).

### DAG treatment did not alter locomotor activity

The different DAG doses did not influence locomotor activity in male rats (Supplementary Fig. 9A–H, 1Q), female rats (Supplementary Fig. 9I–H, 1Q, *n* = 5–6 per treatment group as one male and one female were excluded due to system malfunction) or male mice (F(3,28) = 1.30, *P* = 0.2956; *n* = 8 per treatment group, data not shown).

## Discussion

While previous studies have established that ghrelin and its receptor modulate alcohol-related responses, our study presents novel data demonstrating the regulatory role of DAG in these effects. Collectively, these findings contribute to our knowledge of the ghrelin pathway’s involvement in alcohol-related responses.

The alcohol-drinking experiments revealed that acute DAG treatment decreased both the alcohol intake and preference for alcohol in rats of both sexes. As further evident in both male and female alcohol-drinking rats, the reduced alcohol intake was evident at both 4- and 24-h time points after repeated DAG treatment. Contrarily lower doses of DAG did not influence alcohol drinking, pointing towards a dose-response effect. Together these data add to the present knowledge on DAG, which previously has been found to be opposite to ghrelin. Specifically, DAG suppresses feeding [32] and blocks ghrelin-induced responses [30–34]. On a similar note, DAG’s effects on alcohol intake are similar to that of GHSR antagonists. Specifically, in males systemic GHSR antagonist treatment reduces alcohol intake and also prevents relapse drinking and the motivation to consume alcohol [6–15]. Although these data are the first to point towards a role of DAG in modulating alcohol intake, indirect support is provided as polymorphisms in the *pre-pro-ghrelin* gene, which encodes for DAG and ghrelin, is associated with fewer heavy drinking days and lower average drinks per day [23]. The present DAG data together with studies on ghrelin and GHSR antagonists provide insight into the role of the ghrelin pathway in alcohol-responses. Further support is provided as the recently discovered inverse GHSR agonist, LEAP2, reduces alcohol intake in high alcohol-consuming mice after its central infusion [47].

In the present study, acute administration of DAG to male mice prevented the ability of alcohol to cause locomotor stimulation, dopamine release in NAc shell, and the reward of alcohol in the CPP test. As this dopamine enhancement is associated with alcohol’s rewarding experience and risk for AUD diagnosis [48, 49], we suggest that DAG suppresses the rewarding properties of alcohol, likely contributing to the decline in alcohol intake. These data are in line with other substrates of the ghrelin pathway, as ghrelin increases and GHSR antagonists attenuate reward-related behaviors in male rodents (for review see [4]). While these reward-associated preclinical models are conducted in male mice, the outcome in female mice may diverge and should be evaluated in female subjects in future studies. We further showed that DAG did not ablate the memory of alcohol-induced reward in male mice or alter the monoaminergic neurotransmission in the hippocampus in alcohol-drinking male or female rats. Although the memory of alcohol reward is another important aspect of the AUD process, these data indicate that some alcohol responses but not all are suppressed by DAG. These data are in contrast to that of ghrelin, as ghrelin has been shown to enhance memory function via hippocampal mechanisms [50, 51], and further suggests that ghrelin and DAG act via diverging pathways. The reduction in alcohol intake observed in rats of both sexes is most likely not due to DAG-induced motor dysfunction since the DAG did not alter locomotor activity per se in mice or rats.

While we showed that systemic administration of DAG attenuated alcohol-related responses, the precise mechanisms underlying this effect remain undefined. While limited, our data provide some insight into this as the ex vivo neurochemical data from alcohol-drinking male rats show that systemic DAG enhanced the DOPAC and HVA and tended to elevate dopamine in the VTA. Albeit systemic DAG enters the brain through non-saturable transmembrane diffusion [52], DAG did not affect the number of cFOS-positive neurons of the VTA. Although DAG altered the neurotransmission of the VTA, the neuronal activity of the NAc, the main target area of VTA projections, was unaffected by DAG treatment. Specifically, the number of cFOS-positive cells in NAc was similar between DAG- and vehicle-treated rats. Neither did DAG change monoaminergic neurotransmission in other reward-related areas studied herein. We, therefore, suggest that the altered dopaminergic neurotransmission of the VTA may involve indirect effects rather than direct mechanisms in the VTA. Hence, future studies should explore the site-specific brain detection of fluorescently labeled DAG after its systemic administration. To further identify brain regions affected by DAG, upcoming studies should explore the impact of local DAG infusion into brain areas such as the VTA, on alcohol-related responses. Although the present ex vivo neurochemical data revealed that DAG interacted with dopamine signaling in the VTA and that DAG prevented alcohol from enhancing dopamine in NAc shell, the findings from the recombinant cell system showed that DAG did not inhibit DAT. It should however be considered that DAG may interact with enzymes involved in the production or degradation of dopamine, which will be tested in upcoming studies. Intriguingly, this has been shown for analogs of another gut-brain peptide (GLP-1, semaglutide) [53]. Neither did DAG show any inhibition on SERT or NET, or activity towards 5HT2A, CB1, or µ-opioid receptors. While we excluded DAG’s interactions with some receptors and transporters, the identity of the endogenous receptor for DAG remains elusive [27–31]. While DAG most likely does not interact with GHSR, it may act via an unknown or unidentified receptor which should be defined in future studies. As the binding of DAG was not tested its interaction may remain irrelevant as no discernible activity or inhibition would be manifested. The fact that human rather than rat transporters/receptors were used for these studies should be considered as a tentative confounding factor, however, the impact remains to be determined as the amino acid sequence of human and rat DAG is similar. The inactivity may be attributed also to the tested transporters and receptors which may only respond to small molecules [54, 55]. Although DAG has been found to penetrate the blood-brain barrier [52], peripheral mechanisms should also be considered. One of these is the vagal nerve, as ghrelin’s appetite stimulatory properties are ablated in vagotomized rats [56]. Another possibility is that DAG acts indirectly by changing other substrates of the ghrelin pathway, which in turn would lead to changes in behavioral outcomes. Therefore, the effects of DAG on alcohol-related responses in ghrelin, GHSR, or GOAT (the enzyme converting DAG into ghrelin) knockout models are warranted for future studies. Given the preliminary nature of these mechanistic studies, additional studies are warranted to define neurochemical circuits and receptors affected by DAG in detail.

Although DAG reduced alcohol consumption in rats of both sexes, some sex differences were evident. Specifically, after acute DAG treatment the decline in alcohol consumption was evident at the 24-h time point for males and the 4-h time point for females. Moreover, after repeated DAG treatment the decline was similar at each treatment session for males whereas it escalates over time in females. It should be further noted that DAG treatment did not alter monoaminergic neurotransmission in the VTA or any other investigated areas in alcohol-drinking female rats, whereas DAG elevated dopaminergic neurotransmission in the VTA of males. Further sex-specific differences were demonstrated since the circulating DAG levels were similar in male rats with different alcohol drinking patterns, while they were positively correlated to alcohol consumption in female rats. While the rationale for these sex-dependent discrepancies remains unknown, factors such as different DAG levels may contribute to these effects (for review see [57, 58]). As DAG is eliminated via GOAT conversion to ghrelin, the possibility that the expression of GOAT varies between male and female rats should be considered. In support, estrogen changes the GOAT expression [59, 60]. Moreover, altered ghrelin secretion, stimulated by estrogen [61], may contribute to these sex-specific differences, as ghrelin is converted into DAG. Another contributing factor to the sex-dependent differences could be variations in renal elimination [62], as renal clearance has been suggested to be a major pathway for the elimination of DAG [63, 64]. Another contributing factor may be diverging responses to stress-related ghrelin secretion (for review see [57, 58]). Furthermore, we did not follow the females’ estrous cycle status which should be considered a limitation. However, variation in the estrous cycle appears less likely to influence the outcome as the females’ alcohol intake was stable over time and as the individual variation at each alcohol drinking session was low. Moreover, the possibility that DAG’s mechanistic underpinnings diverge between sexes should be considered, creating a tentative aim for upcoming studies.

While circulating levels of DAG were positively correlated to alcohol consumption in female rats, neither total ghrelin nor obestatin correlated to alcohol consumption in rats of either sex. Supportively, obestatin has not been associated with alcohol drinking before [65]. On the other hand, the correlations between total ghrelin and alcohol drinking are inconclusive. Specifically, some studies show higher total ghrelin at high alcohol intake whereas some studies do not reveal this difference (for review see [66]). While the role of circulating DAG for alcohol intake remains to be elucidated, these correlation findings support the role of systemic DAG as a modulator of alcohol drinking. It is also plausible that females, where DAG levels are positively associated with high alcohol intake, respond differently to DAG treatment than males.

Although a robust suppression of alcohol drinking was found in the DAG-treated rats, no effect on body weight was observed in male or female rats. These data are in contrast to those of ghrelin and GHSR antagonists, which are well known to increase and suppress body weight, respectively (for review see [58, 67]). It is therefore plausible that medications targeting DAG could serve as treatment for regular-weight AUD patients, while agents targeting other targets of the ghrelin pathway might be better options for overweight AUD patients. When it comes to feeding, DAG acutely increased the 24-h food intake in males, whereas repeated treatment overall reduced the 4-h feeding in females. These conflicting data indicate that DAG may influence different systems when injected acutely compared to repeatedly. Specifically, these diverging effects of DAG might be associated with its ability to antagonize ghrelin’s effect as well as act via ghrelin-independent mechanisms as shown before [68]. They further support the sex-divergent differences caused by DAG, as observed in relation to alcohol. Similarly to ghrelin and GHSR antagonists, DAG did not affect the water intake, indicating that DAG robustly reduces alcohol drinking while other consummatory behaviors are less altered by DAG.

In summary, we showed that systemic administration of DAG reduced alcohol intake in male and female rats, as well as mitigated the alcohol-induced reward in male rodents. Taken together with previous studies in which ghrelin enhances and GHSR antagonists suppress alcohol-related responses we provided additional insight into the role of the entire ghrelin pathway.

### Supplementary information

Supplementary Figures

## Data Availability

Data are available upon request.
