# Supplementary material for: Des-acyl ghrelin reduces alcohol intake and alcohol-induced reward in rodents
Source: Transl Psychiatry. 2024 Jul 4;14:277. doi: 10.1038/s41398-024-02996-8 (PMC11224403; doi:10.1038/s41398-024-02996-8)
Supplement: Supplementary file 1 — Supplementary Figures [file 41398_2024_2996_MOESM1_ESM.pptx]

## Slide 1
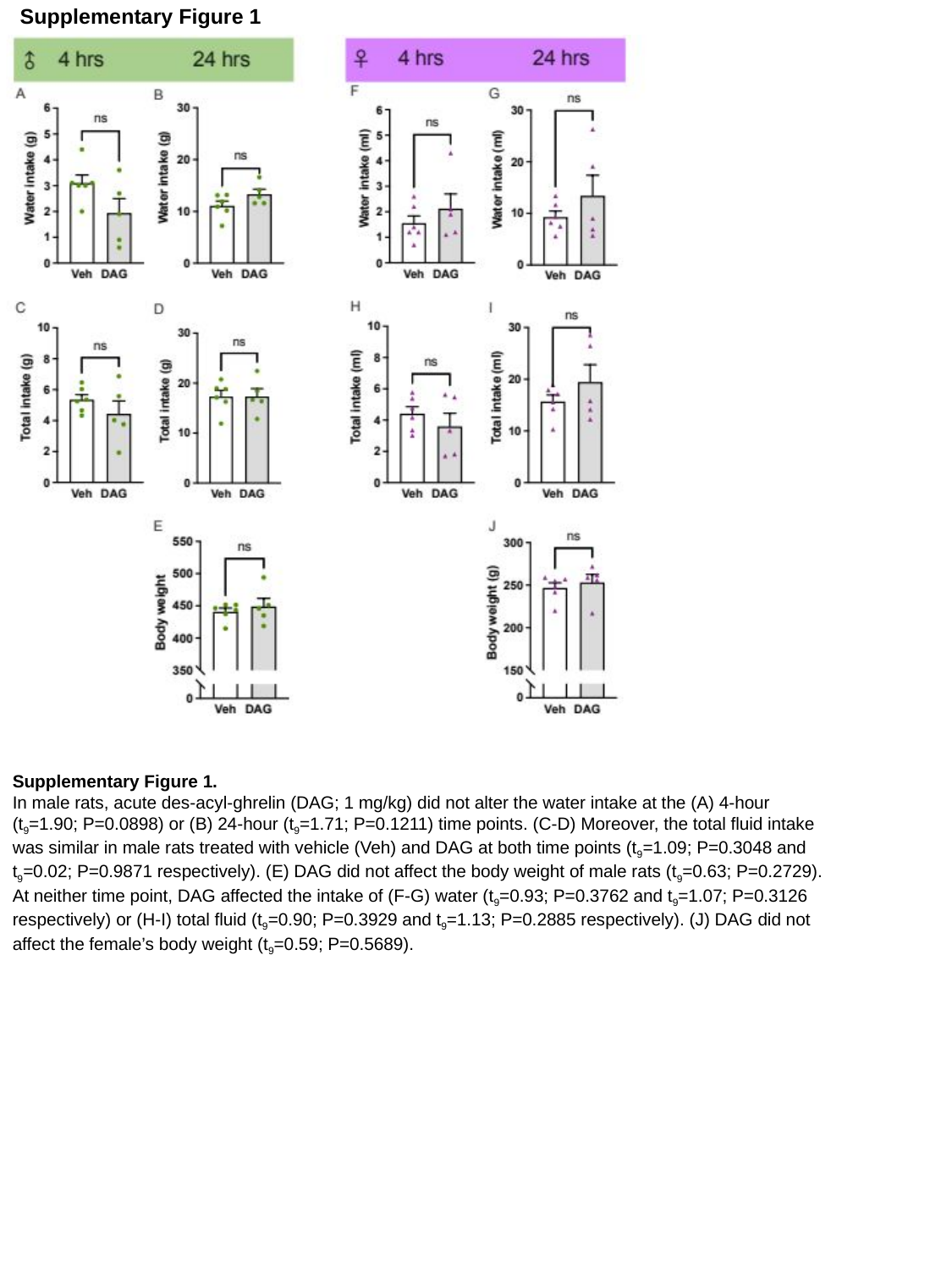

Supplementary Figure 1
Supplementary Figure 1.
In male rats, acute des-acyl-ghrelin (DAG; 1 mg/kg) did not alter the water intake at the (A) 4-hour (t9=1.90; P=0.0898) or (B) 24-hour (t9=1.71; P=0.1211) time points. (C-D) Moreover, the total fluid intake was similar in male rats treated with vehicle (Veh) and DAG at both time points (t9=1.09; P=0.3048 and t9=0.02; P=0.9871 respectively). (E) DAG did not affect the body weight of male rats (t9=0.63; P=0.2729). At neither time point, DAG affected the intake of (F-G) water (t9=0.93; P=0.3762 and t9=1.07; P=0.3126 respectively) or (H-I) total fluid (t9=0.90; P=0.3929 and t9=1.13; P=0.2885 respectively). (J) DAG did not affect the female’s body weight (t9=0.59; P=0.5689).

## Slide 2
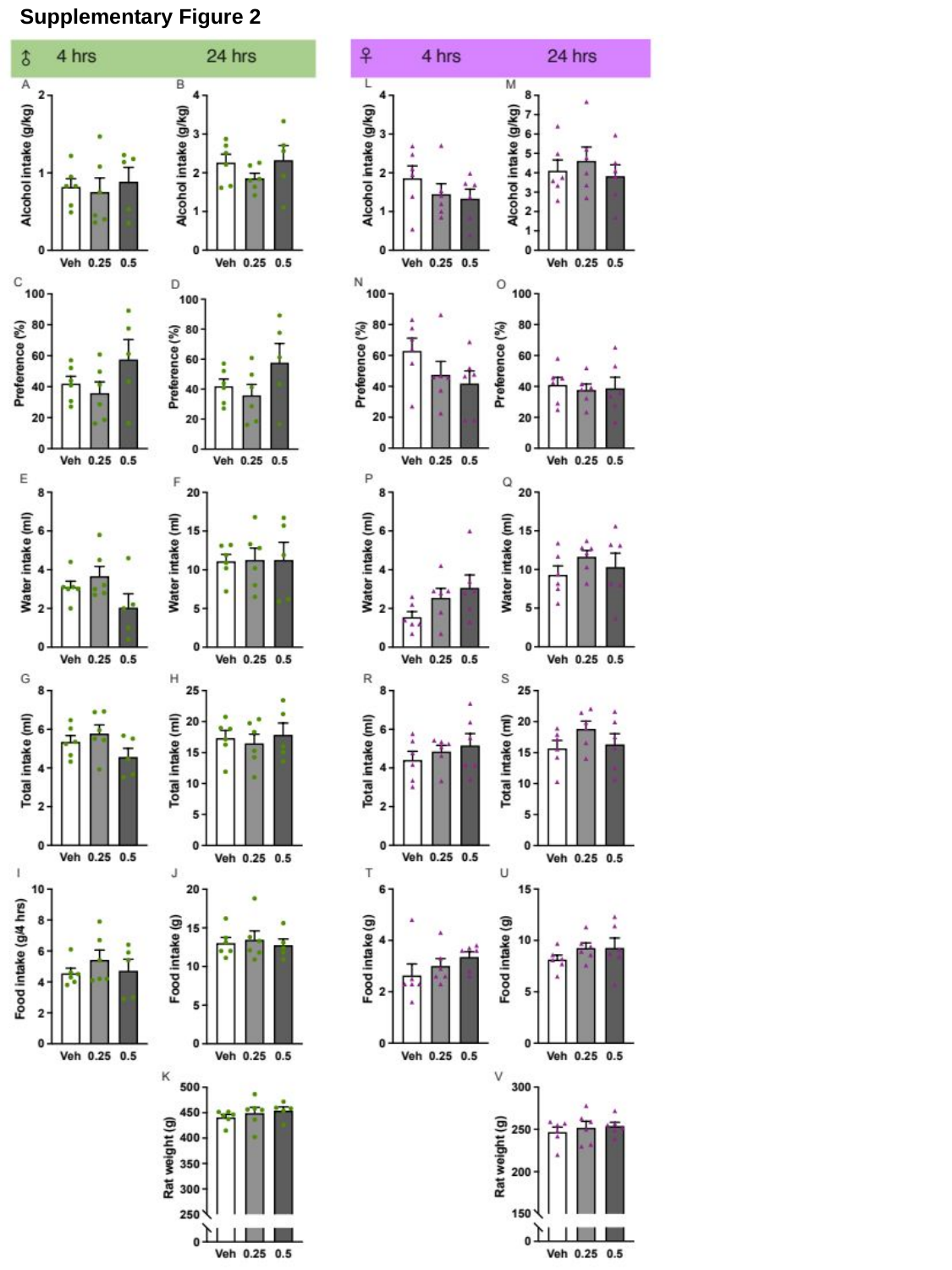

Supplementary Figure 2

## Slide 3
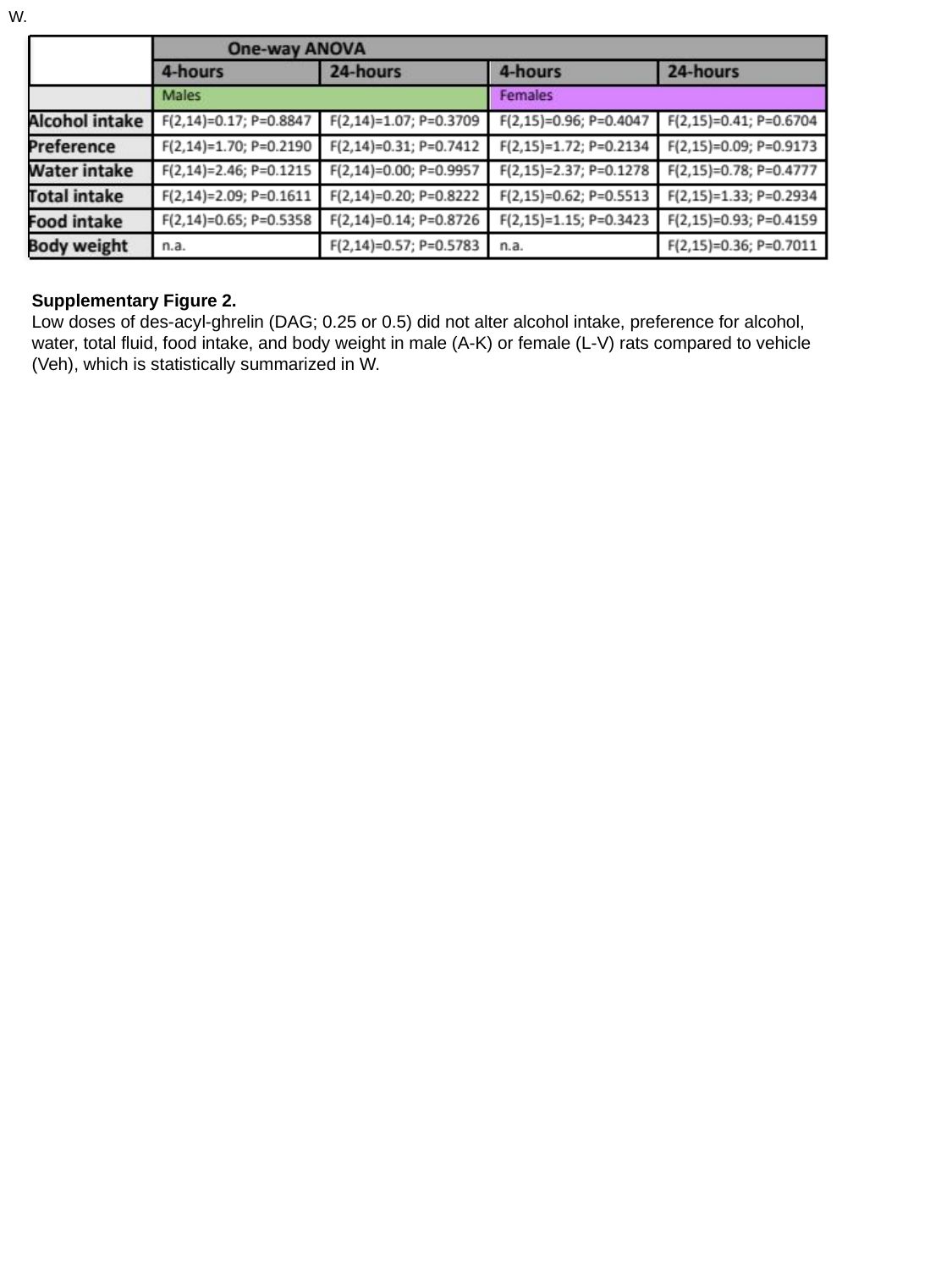

W.
Supplementary Figure 2.
Low doses of des-acyl-ghrelin (DAG; 0.25 or 0.5) did not alter alcohol intake, preference for alcohol, water, total fluid, food intake, and body weight in male (A-K) or female (L-V) rats compared to vehicle (Veh), which is statistically summarized in W.

## Slide 4
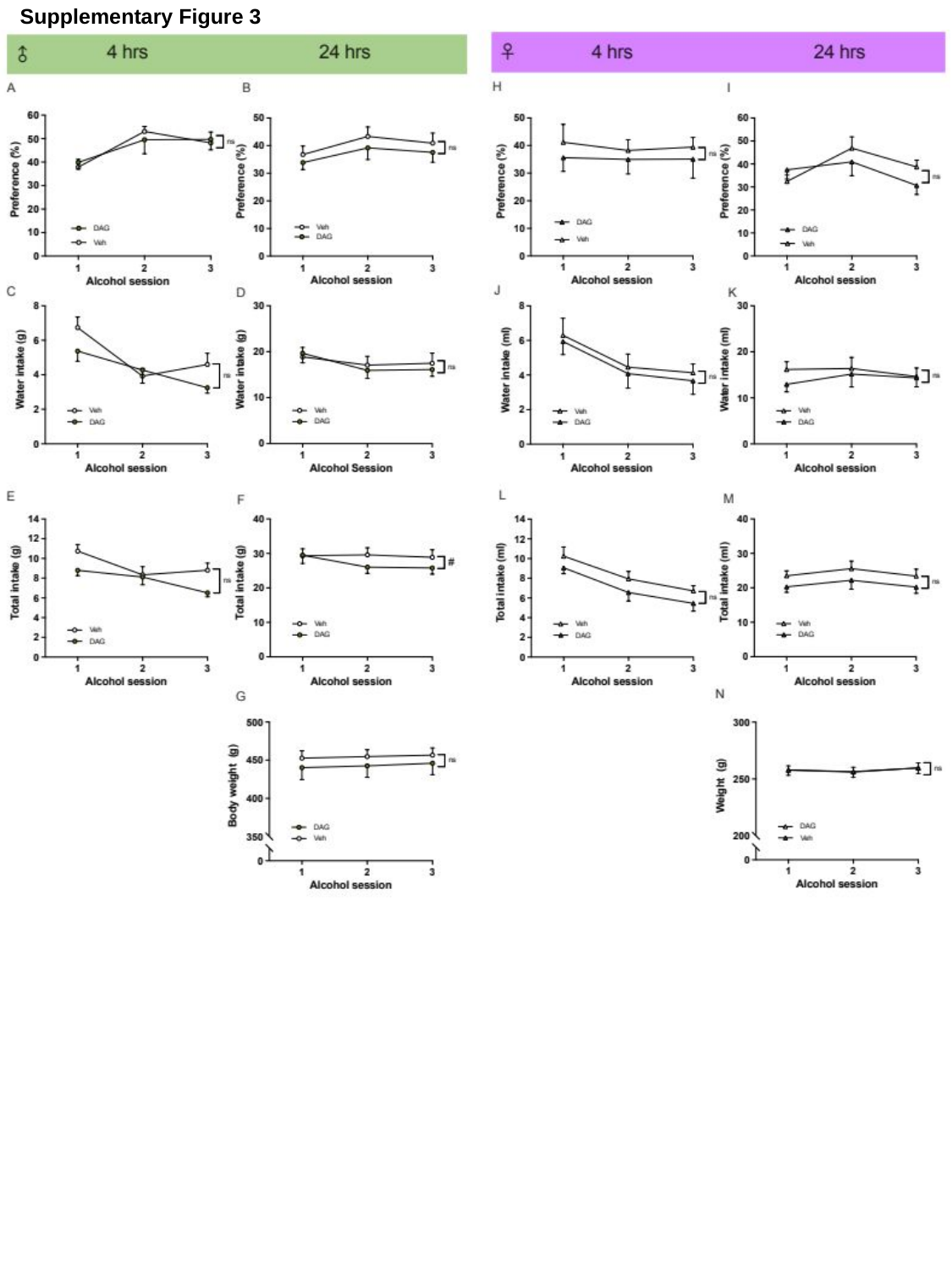

Supplementary Figure 3

## Slide 5
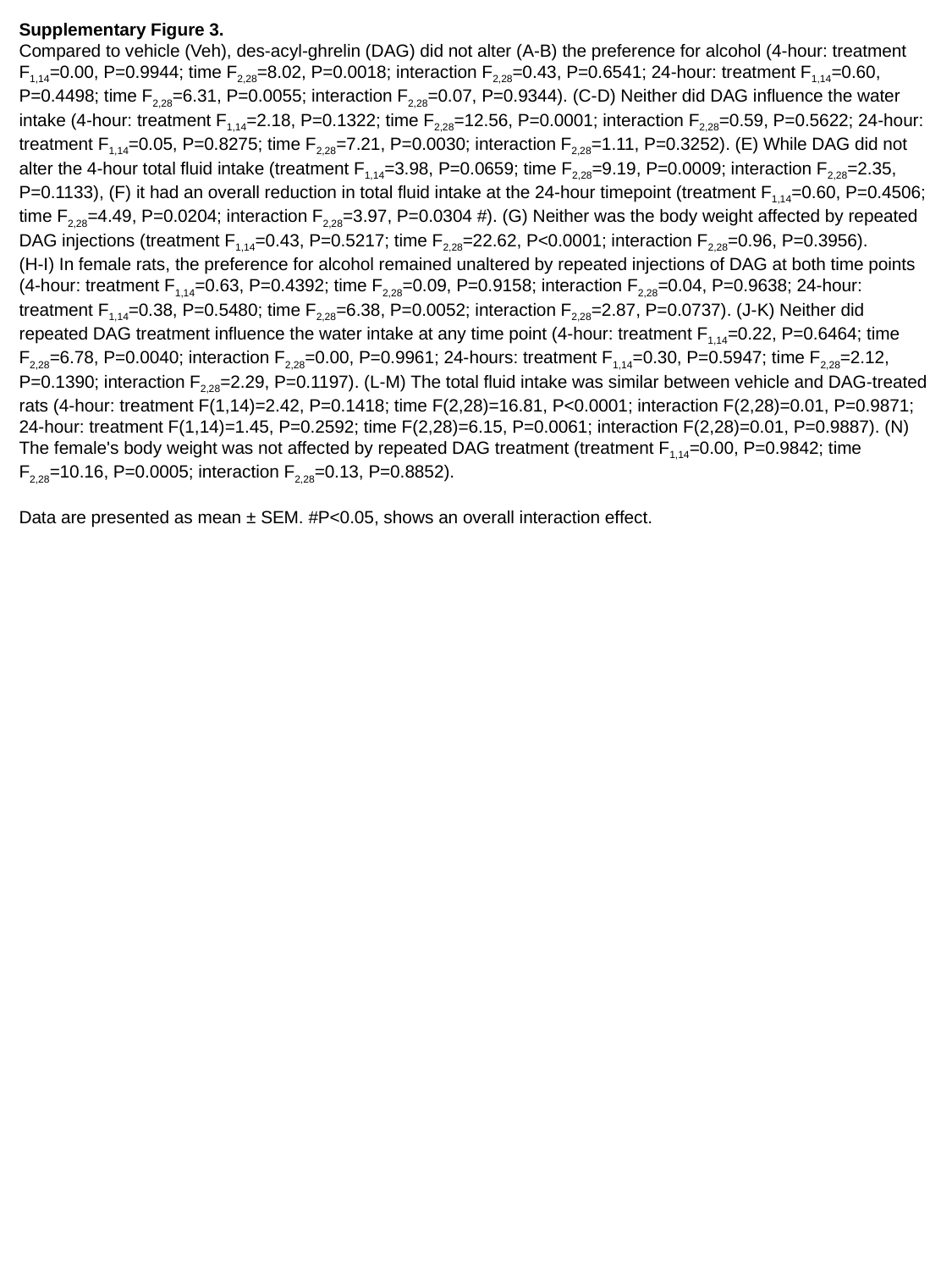

Supplementary Figure 3.
Compared to vehicle (Veh), des-acyl-ghrelin (DAG) did not alter (A-B) the preference for alcohol (4-hour: treatment F1,14=0.00, P=0.9944; time F2,28=8.02, P=0.0018; interaction F2,28=0.43, P=0.6541; 24-hour: treatment F1,14=0.60, P=0.4498; time F2,28=6.31, P=0.0055; interaction F2,28=0.07, P=0.9344). (C-D) Neither did DAG influence the water intake (4-hour: treatment F1,14=2.18, P=0.1322; time F2,28=12.56, P=0.0001; interaction F2,28=0.59, P=0.5622; 24-hour: treatment F1,14=0.05, P=0.8275; time F2,28=7.21, P=0.0030; interaction F2,28=1.11, P=0.3252). (E) While DAG did not alter the 4-hour total fluid intake (treatment F1,14=3.98, P=0.0659; time F2,28=9.19, P=0.0009; interaction F2,28=2.35, P=0.1133), (F) it had an overall reduction in total fluid intake at the 24-hour timepoint (treatment F1,14=0.60, P=0.4506; time F2,28=4.49, P=0.0204; interaction F2,28=3.97, P=0.0304 #). (G) Neither was the body weight affected by repeated DAG injections (treatment F1,14=0.43, P=0.5217; time F2,28=22.62, P<0.0001; interaction F2,28=0.96, P=0.3956).
(H-I) In female rats, the preference for alcohol remained unaltered by repeated injections of DAG at both time points (4-hour: treatment F1,14=0.63, P=0.4392; time F2,28=0.09, P=0.9158; interaction F2,28=0.04, P=0.9638; 24-hour: treatment F1,14=0.38, P=0.5480; time F2,28=6.38, P=0.0052; interaction F2,28=2.87, P=0.0737). (J-K) Neither did repeated DAG treatment influence the water intake at any time point (4-hour: treatment F1,14=0.22, P=0.6464; time F2,28=6.78, P=0.0040; interaction F2,28=0.00, P=0.9961; 24-hours: treatment F1,14=0.30, P=0.5947; time F2,28=2.12, P=0.1390; interaction F2,28=2.29, P=0.1197). (L-M) The total fluid intake was similar between vehicle and DAG-treated rats (4-hour: treatment F(1,14)=2.42, P=0.1418; time F(2,28)=16.81, P<0.0001; interaction F(2,28)=0.01, P=0.9871; 24-hour: treatment F(1,14)=1.45, P=0.2592; time F(2,28)=6.15, P=0.0061; interaction F(2,28)=0.01, P=0.9887). (N) The female's body weight was not affected by repeated DAG treatment (treatment F1,14=0.00, P=0.9842; time F2,28=10.16, P=0.0005; interaction F2,28=0.13, P=0.8852).
Data are presented as mean ± SEM. #P<0.05, shows an overall interaction effect.

## Slide 6
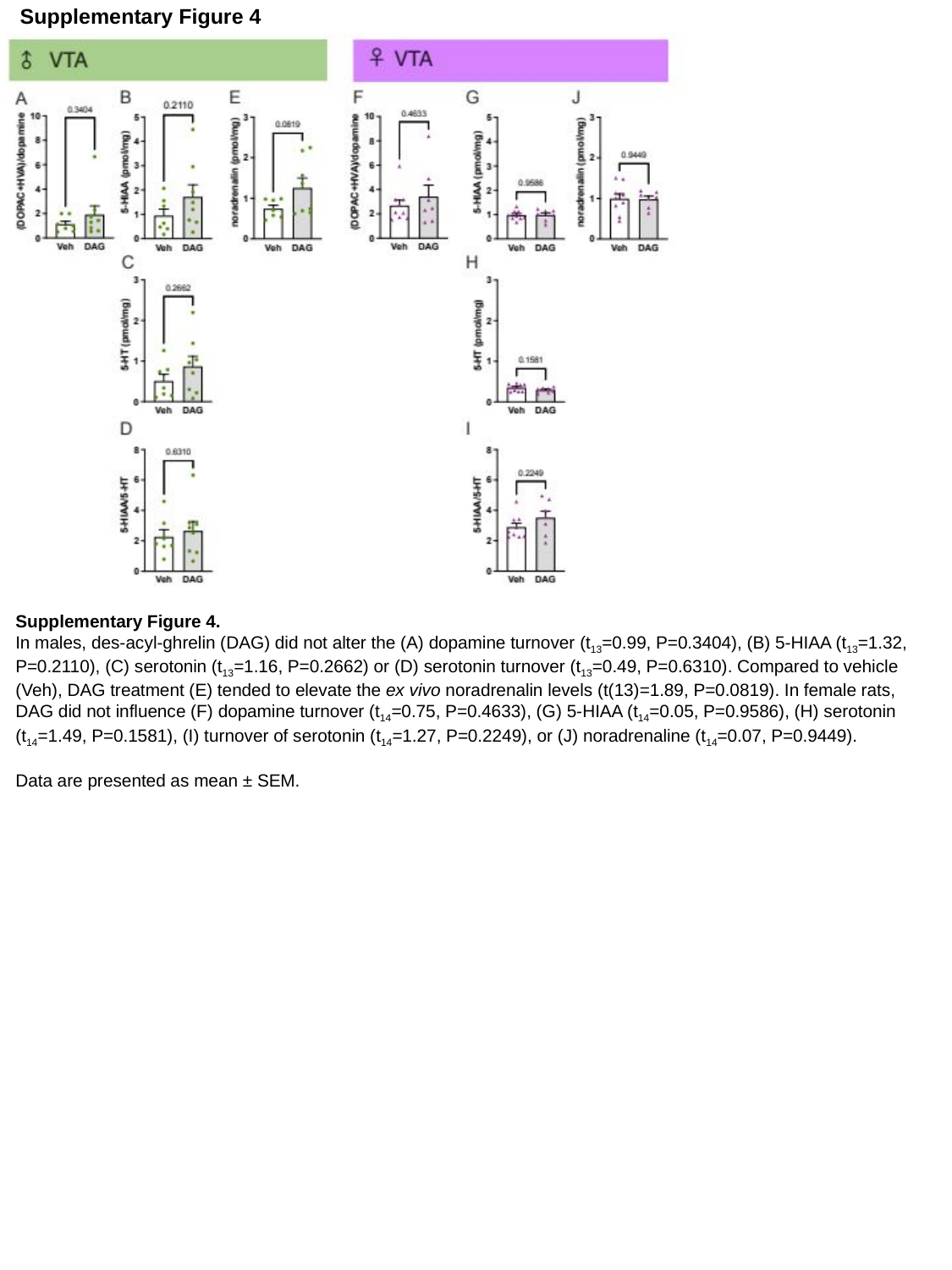

Supplementary Figure 4
Supplementary Figure 4.
In males, des-acyl-ghrelin (DAG) did not alter the (A) dopamine turnover (t13=0.99, P=0.3404), (B) 5-HIAA (t13=1.32, P=0.2110), (C) serotonin (t13=1.16, P=0.2662) or (D) serotonin turnover (t13=0.49, P=0.6310). Compared to vehicle (Veh), DAG treatment (E) tended to elevate the ex vivo noradrenalin levels (t(13)=1.89, P=0.0819). In female rats, DAG did not influence (F) dopamine turnover (t14=0.75, P=0.4633), (G) 5-HIAA (t14=0.05, P=0.9586), (H) serotonin (t14=1.49, P=0.1581), (I) turnover of serotonin (t14=1.27, P=0.2249), or (J) noradrenaline (t14=0.07, P=0.9449).
Data are presented as mean ± SEM.

## Slide 7
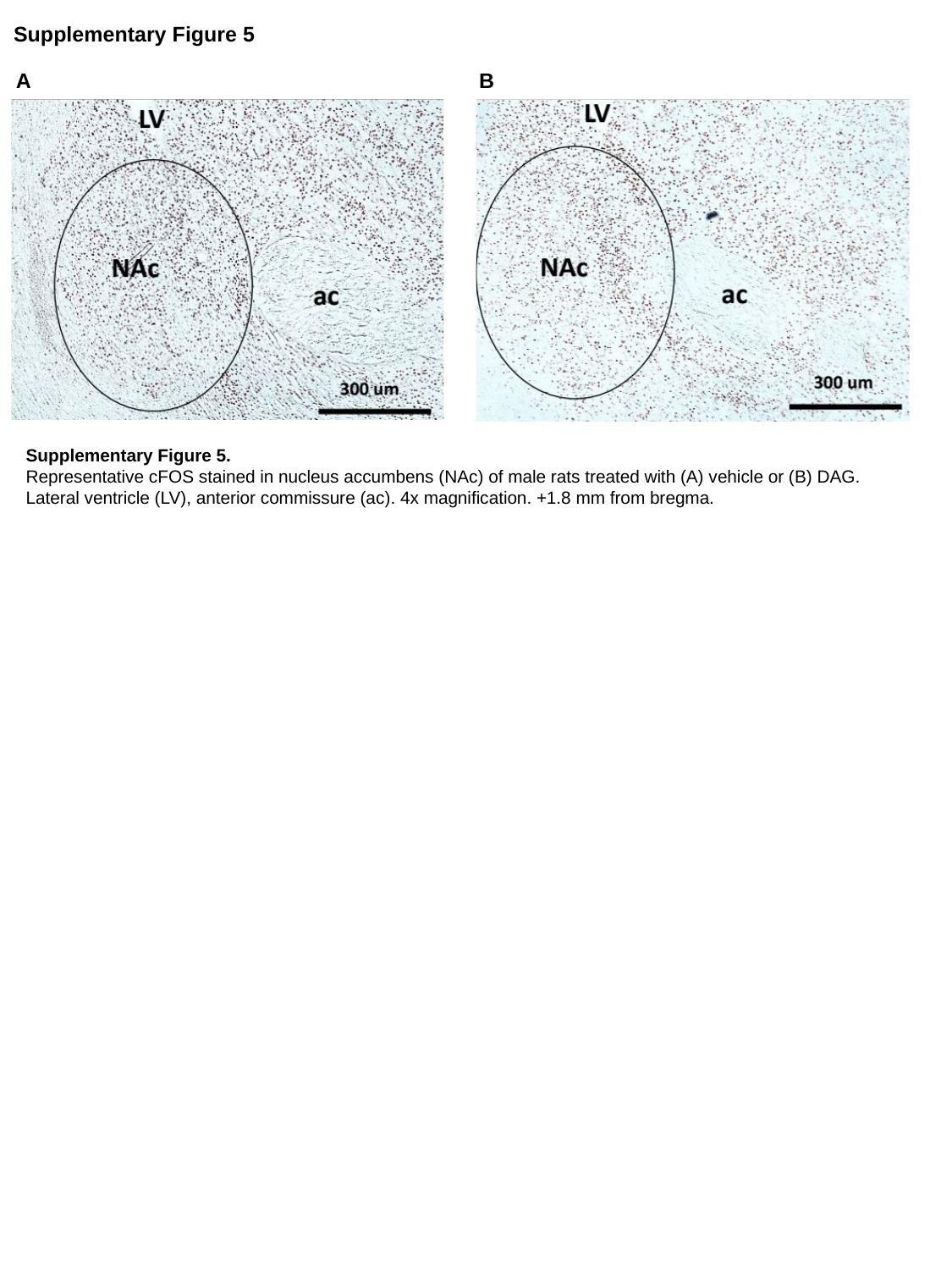

Supplementary Figure 5
A B
Supplementary Figure 5.
Representative cFOS stained in nucleus accumbens (NAc) of male rats treated with (A) vehicle or (B) DAG. Lateral ventricle (LV), anterior commissure (ac). 4x magnification. +1.8 mm from bregma.

## Slide 8
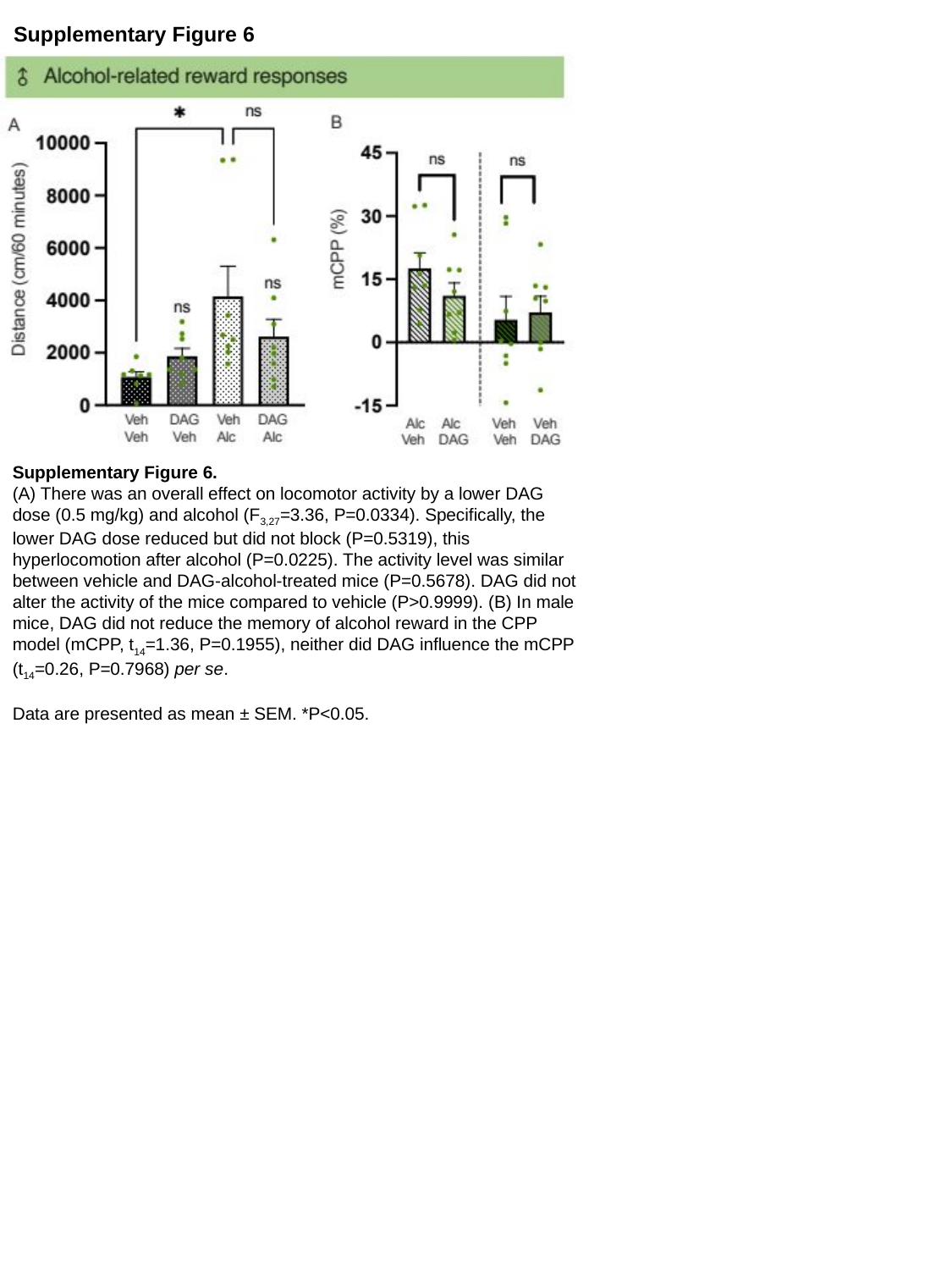

Supplementary Figure 6
Supplementary Figure 6.
(A) There was an overall effect on locomotor activity by a lower DAG dose (0.5 mg/kg) and alcohol (F3,27=3.36, P=0.0334). Specifically, the lower DAG dose reduced but did not block (P=0.5319), this hyperlocomotion after alcohol (P=0.0225). The activity level was similar between vehicle and DAG-alcohol-treated mice (P=0.5678). DAG did not alter the activity of the mice compared to vehicle (P>0.9999). (B) In male mice, DAG did not reduce the memory of alcohol reward in the CPP model (mCPP, t14=1.36, P=0.1955), neither did DAG influence the mCPP (t14=0.26, P=0.7968) per se.
Data are presented as mean ± SEM. *P<0.05.

## Slide 9
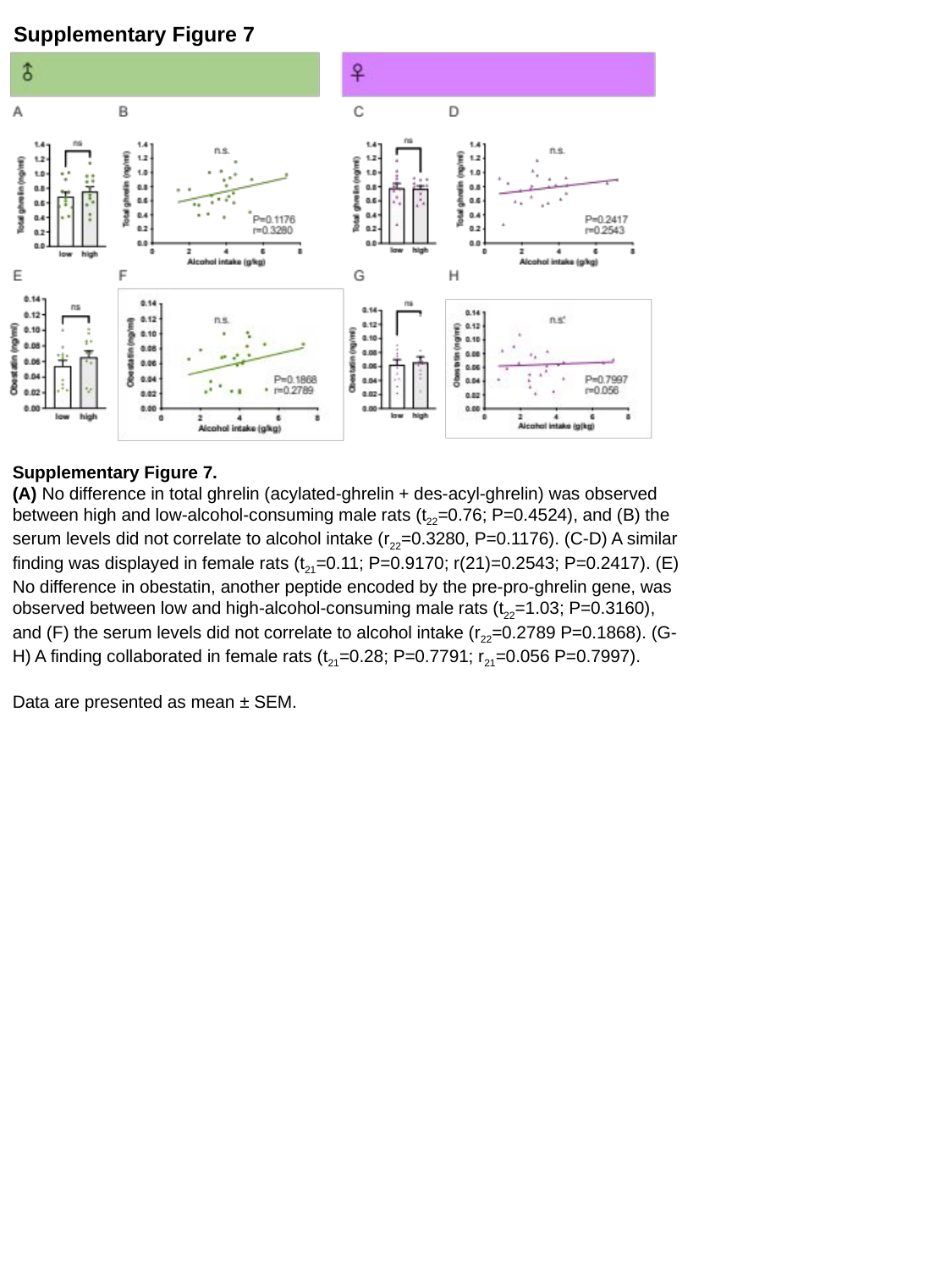

Supplementary Figure 7
Supplementary Figure 7.
(A) No difference in total ghrelin (acylated-ghrelin + des-acyl-ghrelin) was observed between high and low-alcohol-consuming male rats (t22=0.76; P=0.4524), and (B) the serum levels did not correlate to alcohol intake (r22=0.3280, P=0.1176). (C-D) A similar finding was displayed in female rats (t21=0.11; P=0.9170; r(21)=0.2543; P=0.2417). (E) No difference in obestatin, another peptide encoded by the pre-pro-ghrelin gene, was observed between low and high-alcohol-consuming male rats (t22=1.03; P=0.3160), and (F) the serum levels did not correlate to alcohol intake (r22=0.2789 P=0.1868). (G-H) A finding collaborated in female rats (t21=0.28; P=0.7791; r21=0.056 P=0.7997).
Data are presented as mean ± SEM.

## Slide 10
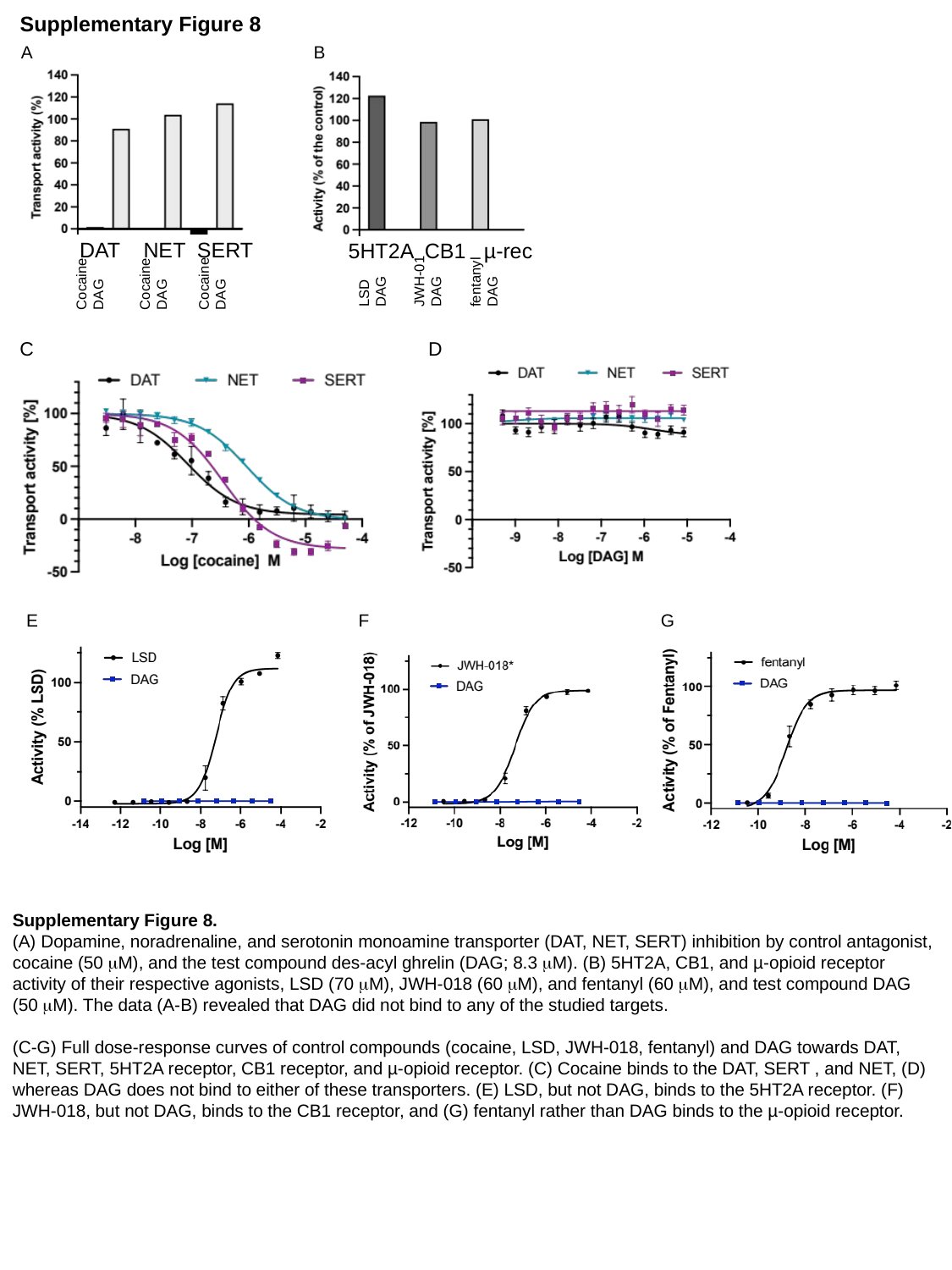

Supplementary Figure 8
A B
DAT NET SERT
Cocaine
DAG
Cocaine
DAG
Cocaine
DAG
5HT2A CB1 µ-rec
JWH-01
DAG
fentanyl
DAG
LSD
DAG
C D
E F G
Supplementary Figure 8.
(A) Dopamine, noradrenaline, and serotonin monoamine transporter (DAT, NET, SERT) inhibition by control antagonist, cocaine (50 μM), and the test compound des-acyl ghrelin (DAG; 8.3 μM). (B) 5HT2A, CB1, and µ-opioid receptor activity of their respective agonists, LSD (70 μM), JWH-018 (60 μM), and fentanyl (60 μM), and test compound DAG (50 μM). The data (A-B) revealed that DAG did not bind to any of the studied targets.
(C-G) Full dose-response curves of control compounds (cocaine, LSD, JWH-018, fentanyl) and DAG towards DAT, NET, SERT, 5HT2A receptor, CB1 receptor, and µ-opioid receptor. (C) Cocaine binds to the DAT, SERT , and NET, (D) whereas DAG does not bind to either of these transporters. (E) LSD, but not DAG, binds to the 5HT2A receptor. (F) JWH-018, but not DAG, binds to the CB1 receptor, and (G) fentanyl rather than DAG binds to the µ-opioid receptor.

## Slide 11
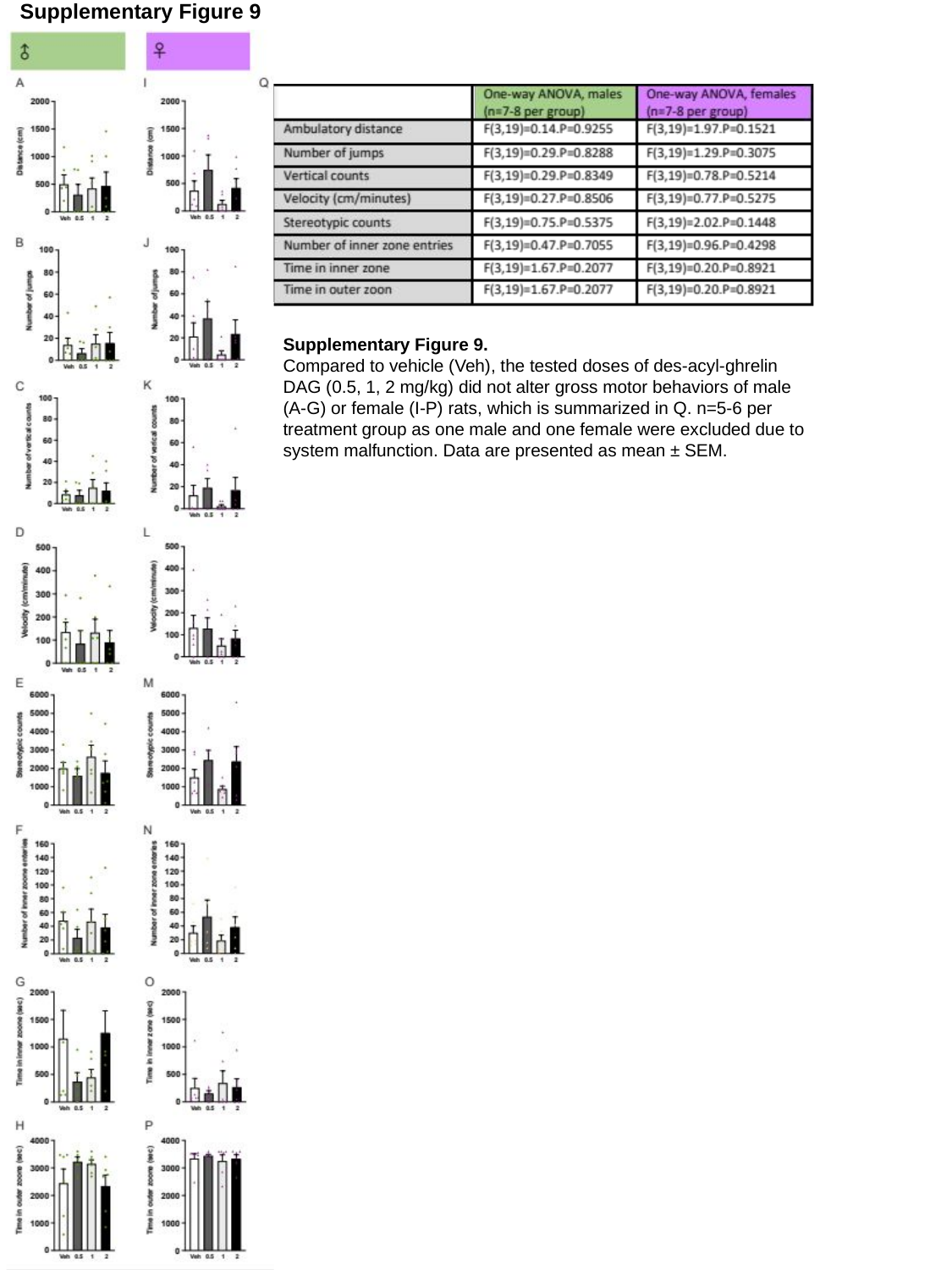

Supplementary Figure 9
Supplementary Figure 9.
Compared to vehicle (Veh), the tested doses of des-acyl-ghrelin DAG (0.5, 1, 2 mg/kg) did not alter gross motor behaviors of male (A-G) or female (I-P) rats, which is summarized in Q. n=5-6 per treatment group as one male and one female were excluded due to system malfunction. Data are presented as mean ± SEM.
